# Targeting PTEN-defined breast cancers with a one-two punch

**DOI:** 10.1186/s13058-015-0566-3

**Published:** 2015-04-08

**Authors:** Leonard B Maggi, Jason D Weber

**Affiliations:** ICCE Institute and Department of Internal Medicine, Division of Molecular Oncology, Siteman Cancer Center, Washington University School of Medicine, St Louis, MO 63110 USA

## Abstract

With tremendous advances in sequencing and analysis in recent years, a wealth of genetic information has become available to identify and classify breast cancer into five main subtypes - luminal A, luminal B, claudin-low, human epidermal growth factor receptor 2-enriched, and basal-like. Current treatment decisions are often based on these classifications, and while more beneficial than any single treatment for all breast cancers, targeted therapeutics have exhibited limited success with most of the subtypes. Luminal B breast cancers are associated with early relapse following endocrine therapy and often exhibit a poor prognosis that is similar to that of the aggressive basal-like breast cancers. Identifying genetic components that contribute to the luminal B endocrine resistant phenotype has become imperative. To this end, numerous groups have identified activation of the phosphatidylinositol 3-kinase (PI3K) pathway as a common recurring event in luminal B cancers with poor outcome. Examining the pathways downstream of PI3K, Fu and colleagues have recreated a human model of the luminal B subtype of breast cancer. The authors were able to reduce expression of phosphatase and tensin homolog (PTEN), the negative regulator of PI3K, using inducible short hairpin RNAs. By varying the expression of PTEN, the authors effectively conferred endocrine resistance and recapitulated the luminal B gene expression signature. Using this system *in vitro* and *in vivo*, they then tested the ability of selective kinase inhibitors downstream of PI3K to enhance current endocrine therapies. A combination of fulvestrant, which blocks ligand-dependent and -independent estrogen receptor signaling, with protein kinase B inhibition was found to overcome endocrine resistance. These findings squarely place PTEN expression levels at the nexus of luminal B breast cancers and indicates that patients with PTEN-low estrogen receptor-positive tumors might benefit from combined endocrine and PI3K pathway therapies.

The phosphatidylinositol 3-kinase (PI3K) pathway has been the focus of intense pre-clinical and clinical investigation due to its high frequency of alteration in human cancers. Luminal B breast cancers are no exception and have proven exceedingly difficult to treat clinically. With current endocrine therapies having limited success in luminal B breast cancer, Fu and colleagues in *Breast Cancer Research* [1] have tested several combinations of kinase inhibitors with antiestrogen treatment to determine if this one-two punch is more effective at inhibiting breast cancer cell growth. Luminal B breast cancers typically exhibit activation of the PI3K pathway and have a worse outcome [2,3]. While luminal B tumors present a lower frequency of *PIK3CA* mutations than luminal A tumors, they do display a greater frequency of phosphatase and tensin homolog (PTEN) aberrations [4]. Subsequently, these PTEN-reduced tumor cells display greater PI3K pathway activation [2] and resistance to endocrine therapies [5-8].

In the present study, Fu and colleagues [1] generated human estrogen receptor-positive (ER+) breast cancer cell lines that contained inducible PTEN short hairpin RNAs, thus allowing them to dial down the expression of PTEN expression to varying levels. Moderate decreases in PTEN expression resulted in the hyperactivation of the PI3K pathway and a concomitant gene expression change most similar to luminal B breast cancers [9]. Notably, these changes were readily apparent with only moderate decreases in PTEN expression, arguing that complete loss of *PTEN*, as observed in many ER-negative breast cancers [10], is not requisite to elicit PI3K pathway hyperactivation in ER+ cells. Reduction in PTEN expression rendered these ER+ cells resistant to antiestrogen treatments, with the authors again dialing PTEN expression downward to achieve more resistance *in vitro* and *in vivo*. These striking results provide the first substantial evidence that PTEN expression is intimately linked to antiestrogen sensitivity.

While targeted therapies for luminal B breast cancers have not yet become clinically apparent, the authors’ initial findings point towards heightened PI3K signaling as a possible key contributor to aberrant proliferation and tumorigenesis. Given their results linking PTEN levels and antiestrogen resistance, the authors sought to combine inhibition of PI3K and mitogen-activated protein kinase pathway components with antiestrogen treatment to elicit an anti-tumor response. Employing antiestrogen treatment with clinically relevant concentrations of mammalian target of rapamycin (mTOR), protein kinase B (AKT) and mitogen-activated protein kinase kinase inhibitors led to significant attenuation of cell growth. Furthermore, when antiestrogen treatment was combined with mTOR and AKT inhibitors, cell growth was massively suppressed and apoptosis was increased, strongly indicating a synergistic effect of antiestrogen treatment alongside mTOR and AKT inhibition. Although the levels of PTEN and the antiestrogen used dictated the extent of the response *in vitro*, the *in vivo* combination of fulvestrant with an AKT inhibitor significantly accelerated tumor regression (three-fold) compared with either inhibitor alone. While this study did not include the use of direct PI3K pharmacological inhibitors, one might also expect that a broad spectrum anti-PI3K agent might also prove efficacious in combination with fulvestrant.

This study is consistent with previous work that showed PTEN loss and *PIK3CA* mutation were not mutually exclusive [11] and builds on evidence that *PIK3CA* mutations do not segregate with high or low PTEN-expressing tumors [10]. Moreover, *PIK3CA* mutations are associated with a better outcome in ER+ breast cancer while PTEN deficiency is correlated with a poor prognosis [2,10]. However, these initial studies were somewhat handicapped by their yes-or-no assessment of PTEN expression. The current study implies that small changes in PTEN expression are sufficient to elicit a growth advantage and treatment-resistance phenotype to breast cancer cells. Thus, regardless of *PIK3CA* status, PTEN levels could be used as a predictive marker for endocrine therapy. However, a clear limitation of the current study is its heavy reliance on established breast cancer cell lines. Additional work in physiological settings (for example, patient-derived xenografts) would provide further validation that this might be a viable clinical strategy. While implementing a PTEN detection strategy and expression level cutoff clinically could prove challenging, the utility of estrogen deprivation in combination with AKT inhibitors holds tremendous promise in effectively treating ER+ tumors with reduced PTEN.

## Abbreviations

AKT: Protein kinase B
ER+: Estrogen receptor-positive
mTOR: Mammalian target of rapamycin
PI3K: Phosphatidylinositol 3-kinase
PTEN: Phosphatase and tensin homolog
